# Fat-free mass index and apixaban exposure in frail hospitalized older adults: An exploratory study

**DOI:** 10.1016/j.tjfa.2026.100201

**Published:** 2026-09-03

**Authors:** Tancrède de Jerphanion, Olivier Brière, Jennifer Gautier, Alexis Bourgeais, Myriam Mellou, Léa Bourreau, Cédric Annweiler, Mathieu Corvaisier

**Affiliations:** a UNIV ANGERS, School of Medicine, Health Faculty, University of Angers, 49045 Angers, France; b UNIV ANGERS, School of Pharmacy, Health Faculty, University of Angers, 49045 Angers, France; c UNIV ANGERS, UPRES EA 4638, University of Angers, Angers, France; d Department of Geriatric Medicine and Memory Clinic, Research Center on Autonomy and Longevity, University Hospital, Angers, France; e Department of Pharmacy, Angers University Hospital, 49933 Angers, France; f Gérontopôle Autonomie Longévité des Pays de la Loire, France; g Robarts Research Institute, Department of Medical Biophysics, Schulich School of Medicine and Dentistry, the University of Western Ontario, London, ON, Canada

**Keywords:** Apixaban, Older adults, Sarcopenia, Fat-free mass index, Direct oral anticoagulants, Frailty, Pharmacokinetics, Bioelectrical impedance analysis

## Abstract

**Background:**

Reduced muscle mass may influence creatinine-based renal estimates and direct oral anticoagulant exposure in frail older adults.

**Objectives:**

To explore the relationship between fat-free mass index (FFMI) and apixaban trough concentrations in hospitalized older adults.

**Methods:**

In this retrospective study, FFMI was assessed by bioelectrical impedance analysis in patients aged ≥75 years treated with apixaban. FFMI was considered a body-composition marker, not a diagnostic criterion for sarcopenia.

**Results:**

Among 305 screened patients, 43 receiving apixaban 5 mg twice daily with compliant trough sampling were included. Mean age was 83.6 ± 5.8 years. FFMI correlated inversely with apixaban concentrations in the unadjusted analysis (Spearman’s ρ = −0.34, *p* = 0.024). In univariable linear regression, lower FFMI was associated with higher apixaban concentrations (β = −8.64 ng/mL per kg/m², 95% CI −16.15 to −1.13; *p* = 0.025), but this association was not confirmed after adjustment for sex and serum creatinine (adjusted β = −6.77, 95% CI −15.20 to 1.65; *p* = 0.112) or in the sensitivity analysis including both dose groups (*n* = 82; ρ = −0.06, *p* = 0.575). Body weight, BMI, and creatinine-based renal estimates were not significantly associated with apixaban concentrations.

**Conclusions:**

An inverse relationship between FFMI and apixaban trough concentrations was observed in the unadjusted primary analysis but was not confirmed after multivariable adjustment or in the sensitivity analysis. This preliminary signal should be considered hypothesis-generating and requires confirmation in larger prospective studies.

## 1 Introduction

Direct oral anticoagulants (DOACs) are widely prescribed in older adults for atrial fibrillation and venous thromboembolism [1]. Their fixed dosing regimens and the absence of routine therapeutic drug monitoring have facilitated their broad use. However, physiological changes, comorbidities, and altered body composition may substantially affect drug exposure and safety in this heterogeneous population [2,3].

Sarcopenia and loss of muscle mass are common in frail older adults and affect serum creatinine, the main biomarker used to estimate renal function [4]. Because creatinine production depends on muscle mass, creatinine-based equations may overestimate glomerular filtration rate in sarcopenic patients and lead to inappropriate dosing of renally cleared drugs. This issue is particularly relevant for DOACs, whose dosing partly relies on renal function estimates [5].

Muscle mass may also influence DOAC activity. Bendayan et al. reported an association between lower lean body mass and higher DOAC activity in older patients with atrial fibrillation [6]. However, the relationship between body composition, drug exposure, and creatinine-based renal estimates remains insufficiently explored in frail hospitalized older adults.

Apixaban is frequently prescribed in older patients. Although its pharmacokinetics are influenced by renal function, age, and body weight, substantial interindividual variability persists [7]. Current dosing criteria based largely on serum creatinine and body weight may therefore incompletely capture the physiological heterogeneity of frail older adults.

Fat-free mass index (FFMI), derived from bioelectrical impedance analysis, reflects fat-free mass normalized for height and provides a clinically accessible marker of body composition [8]. It may help identify reduced muscle mass but does not establish sarcopenia, which also requires assessment of muscle strength and physical performance, and it is not a surrogate for the broader concept of frailty. Its relationship with DOAC exposure and renal function interpretation remains poorly understood.

We hypothesized that low FFMI, as a marker of reduced muscle mass, could be associated with higher apixaban trough concentrations through two non-exclusive mechanisms: overestimation of renal function due to reduced creatinine production and a lower apparent volume of distribution associated with reduced lean mass. The study was not designed to distinguish between these mechanisms. Trough concentrations were selected because predose sampling is feasible in routine care and is relevant to drug elimination and accumulation, whereas peak assessment requires tightly standardized post-dose timing. We therefore explored the relationship between FFMI and apixaban trough concentrations in hospitalized older adults.

## 2 Methods

### 2.1 Study design

We conducted a retrospective observational study using routine-care data from frail older adults treated with apixaban in a tertiary-hospital geriatric acute care unit.

In routine practice, BIA may be contraindicated in patients with implanted cardiac devices or unreliable in the presence of fluid overload, while apixaban concentrations may be difficult to interpret when sampling does not meet predefined trough conditions. These constraints influenced patient selection but were not assessed as formal feasibility outcomes.

### 2.2 Participants

All patients aged ≥75 years treated with apixaban and hospitalized in the unit between May 2024 and May 2025 were screened.

Patients were included if they met the following criteria: (1) Treatment with apixaban at a stable dose for at least five days (standard or reduced dose); (2) Availability of both apixaban trough concentration measurement and body composition assessment allowing the calculation of fat-free mass index (FFMI).

Patients were excluded if: (1) Apixaban plasma sampling did not meet the operational timing criteria described below or could not be reliably interpreted because the timing of drug administration or blood collection was uncertain; (2) Body composition assessment was unavailable or considered unreliable due to clinical or technical reasons, such as contraindications to bioelectrical impedance analysis (e.g., implanted cardiac devices) or conditions affecting measurement accuracy (e.g., peripheral edema); (3) Key clinical or biological data required for the analyses were missing.

### 2.3 Clinical and demographic data

Demographic, clinical, and biological data were retrospectively extracted from electronic records, including age, sex, height, weight, BMI, apixaban indication and dose, comorbidities, concomitant medications, serum creatinine, and albumin.

Comorbidity burden was assessed using the Charlson Comorbidity Index (CCI) [9], frailty using the Clinical Frailty Scale (CFS) [10], and functional status using Activities of Daily Living (ADL) and Instrumental Activities of Daily Living (IADL) scales [11,12]. Renal function was assessed using serum creatinine and Cockcroft–Gault, 2021 CKD-EPI, MDRD, and BIS-1 equations [[13], [14], [15], [16]]. For the exploratory regression analysis, severe undernutrition was operationally defined as a serum albumin concentration <30 g/L. Polypharmacy was defined as more than four chronic medications [17].

### 2.4 Assessment of body composition and FFMI

Body composition was assessed in routine care using a multifrequency BIA device (Bodystat QuadScan 4000, Bodystat Ltd, UK) according to the manufacturer’s instructions [18]. Trained nursing staff or physicians performed measurements with patients supine whenever feasible, using a tetrapolar electrode configuration on the distal extremities of the right side. FFMI was calculated as fat-free mass divided by height squared (kg/m²) and analyzed as a continuous variable.

### 2.5 Assessment of apixaban trough concentrations

Apixaban plasma concentrations were measured in routine clinical practice at the discretion of the treating team; the specific clinical indication was not systematically recorded. Trough concentrations were selected because predose sampling was more readily standardized than peak sampling and was consistent with the study hypothesis regarding elimination and accumulation.

Blood-sampling and apixaban-administration times were manually verified in the source medical records for all patients with both apixaban concentration and BIA-derived FFMI available. Sampling was operationally classified as compliant when blood was collected at the end of the dosing interval before the next administration or within 30 min after administration. Samples obtained more than 30 min after administration or with uncertain timing were classified as non-compliant. Because this operational definition included a limited number of early post-dose samples, the measurements should be interpreted as trough or near-trough concentrations. Concentrations were measured using an apixaban-calibrated chromogenic anti-factor Xa assay.

### 2.6 Definition of exposure groups

Apixaban concentrations were analyzed continuously and descriptively categorized. Concentrations >230 ng/mL, corresponding to the upper expected on-therapy trough range for apixaban 5 mg twice daily, were considered exploratory indicators of higher exposure rather than toxicity thresholds [19,20]. Patients in the upper observed quartile were also compared with the remaining population.

### 2.7 Statistical analysis

Continuous variables were reported as mean ± standard deviation or median [interquartile range], and categorical variables as n (%). Spearman correlations assessed relationships between apixaban trough concentrations, FFMI, and renal estimates. Linear regression was used to explore factors associated with apixaban concentrations. A parsimonious multivariable model specified a priori included FFMI, sex, and serum creatinine, selected for biological plausibility and clinical relevance. Serum creatinine was preferred to a derived renal equation to avoid redundancy. Higher-exposure groups were compared using Mann–Whitney U and chi-square or Fisher’s exact tests, as appropriate. Renal function was estimated using several creatinine-based equations, including Cockcroft–Gault, CKD-EPI, MDRD, and BIS-1 equations. Correlations between these estimations and apixaban concentrations were explored descriptively.

The primary analysis was restricted to patients receiving apixaban 5 mg twice daily. Baseline characteristics were compared between patients receiving 5 mg and 2.5 mg twice daily, and a sensitivity analysis included both dose groups. Among patients with both BIA and apixaban measurements, characteristics were also compared according to sampling compliance.

No formal sample-size calculation was performed, and all eligible patients were included. No adjustment for multiple comparisons was applied; all *p*-values were considered nominal. A two-sided *p*-value <0.05 identified exploratory signals. Analyses were performed using R version 4.6.1.

### 2.8 Ethics

The study was conducted in accordance with the Declaration of Helsinki. The study protocol was approved by the local ethics committee under reference 2025-114. Given the retrospective design and use of routine care data, patient consent was not required according to French regulations.

## 3 Results

### 3.1 Study population

**Fig. 1:**
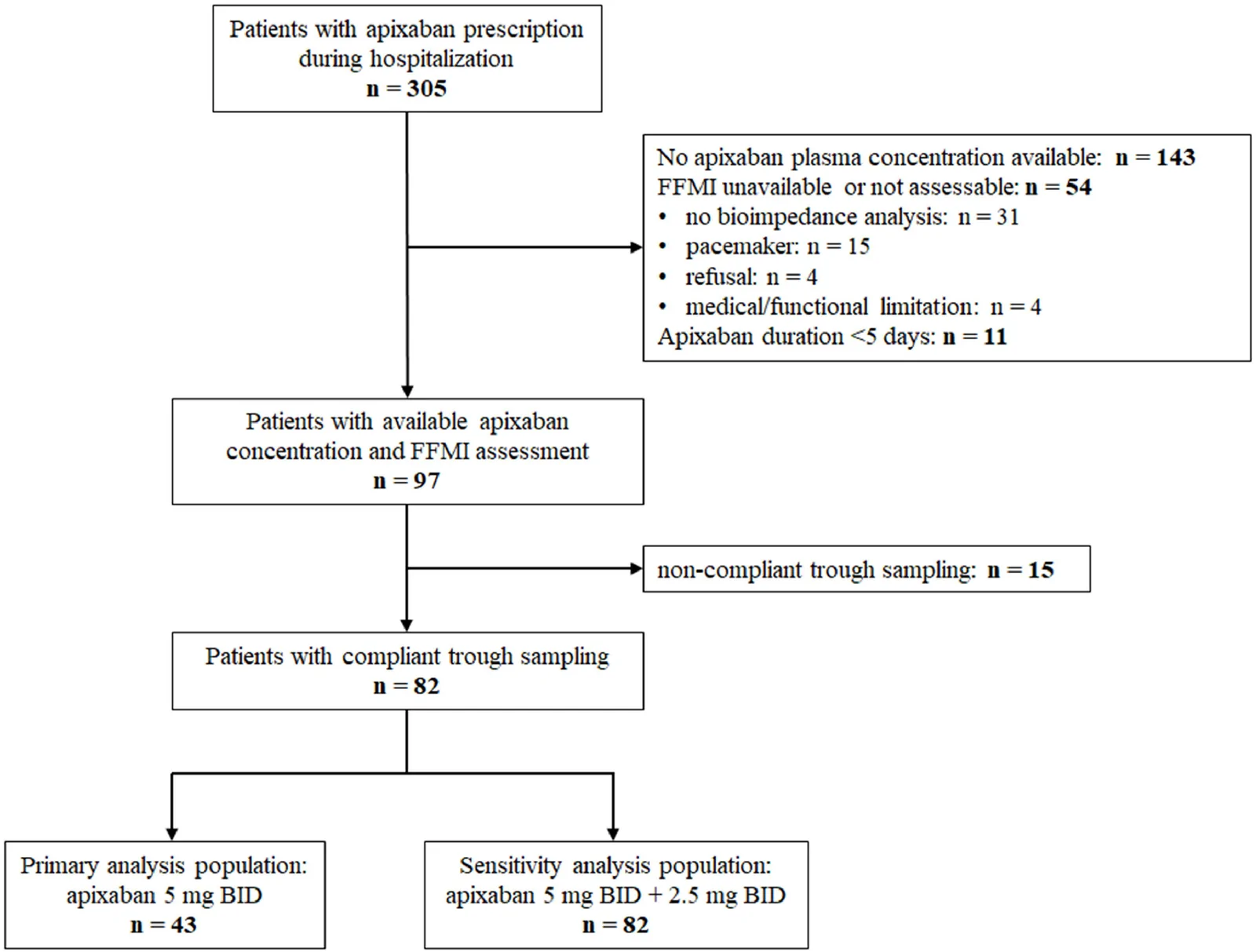
Flow chart of patient selection and study population constitution.

BID: twice daily; FFMI: fat-free mass index.

Among 305 hospitalized patients aged ≥75 years receiving apixaban, 97 had both an apixaban plasma concentration measurement and an assessable BIA-derived FFMI (Fig. 1). The main reasons for exclusion were the absence of an apixaban plasma concentration measurement and unavailable or non-assessable BIA measurements.

Among the 97 eligible patients, 82 had compliant trough sampling conditions, including 43 patients receiving apixaban 5 mg twice daily and 39 receiving 2.5 mg twice daily. The 43 patients receiving 5 mg twice daily constituted the primary analysis population, while all 82 patients were included in the sensitivity analysis.

Among the 97 patients with both measurements available, no statistically significant differences were observed between patients with compliant (*n* = 82) and non-compliant sampling (*n* = 15) for the available demographic, anthropometric, geriatric, body-composition, or renal variables (Supplementary Table 2).

Among patients with compliant sampling, those receiving apixaban 2.5 mg twice daily were older, more frequently female, more frail, and had a higher comorbidity burden than those receiving 5 mg twice daily. They also had lower ADL scores, body weight, BMI, FFMI, and CKD-EPI 2021 estimates, and higher serum creatinine concentrations. Apixaban trough concentrations did not differ significantly between the dose groups (125.2 ± 81.3 vs 140.5 ± 79.0 ng/mL; *p* = 0.281) (Supplementary Table 3).

### 3.2 Patient characteristics

Characteristics of the primary analysis population are summarized in Table 1. Mean age was 83.6 ± 5.8 years, mean Clinical Frailty Scale score was 5.2 ± 1.0, and mean Charlson Comorbidity Index was 7.8 ± 2.4. Reasons for hospital admission were heterogeneous.

**Table 1:** Characteristics of the primary analysis population (*n* = 43).

Variable	Overall population
**Demographic and geriatric characteristics**	
Age, years	83.6 ± 5.8
Female sex, n (%)	15 (34.9%)
Clinical Frailty Scale	5.2 ± 1.0
Charlson Comorbidity Index	7.8 ± 2.4
ADL score	4.3 ± 1.6
IADL score	1.5 ± 1.4
**Anthropometric and body composition parameters**	
Weight, kg	76.1 ± 16.3
BMI, kg/m²	27.7 ± 6.5
FFMI, kg/m²	16.8 ± 3.1
**Renal function assessment**	
Serum creatinine, µmol/L	75.2 ± 24.5
eGFR CKD-EPI 2021, mL/min/1.73 m²	79.6 ± 16.4
eGFR MDRD, mL/min/1.73 m²	86.2 ± 28.9
Estimated creatinine clearance by Cockcroft–Gault, mL/min	67.4 [51.1–86.1]
eGFR BIS-1, mL/min/1.73 m²	64.8 ± 17.6
**Apixaban treatment and exposure**	
Apixaban trough concentration, ng/mL	140.5 ± 79.0
Patients above expected trough range (>230 ng/mL), n (%)	4 (9.3%)

Values are presented as mean ± standard deviation, median [interquartile range], or n (%), as appropriate. The upper expected on-therapy trough concentration for apixaban 5 mg twice daily was defined as >230 ng/mL. ADL: Activities of Daily Living; BIS-1: Berlin Initiative Study 1 equation; BMI: body mass index; CKD-EPI: Chronic Kidney Disease Epidemiology Collaboration; eGFR: estimated glomerular filtration rate; FFMI: fat-free mass index; IADL: Instrumental Activities of Daily Living; MDRD: Modification of Diet in Renal Disease.

Mean FFMI was 16.8 ± 3.1 kg/m² and mean BMI was 27.7 ± 6.5 kg/m².

### 3.3 Association between FFMI and apixaban concentrations

**Fig. 2:**
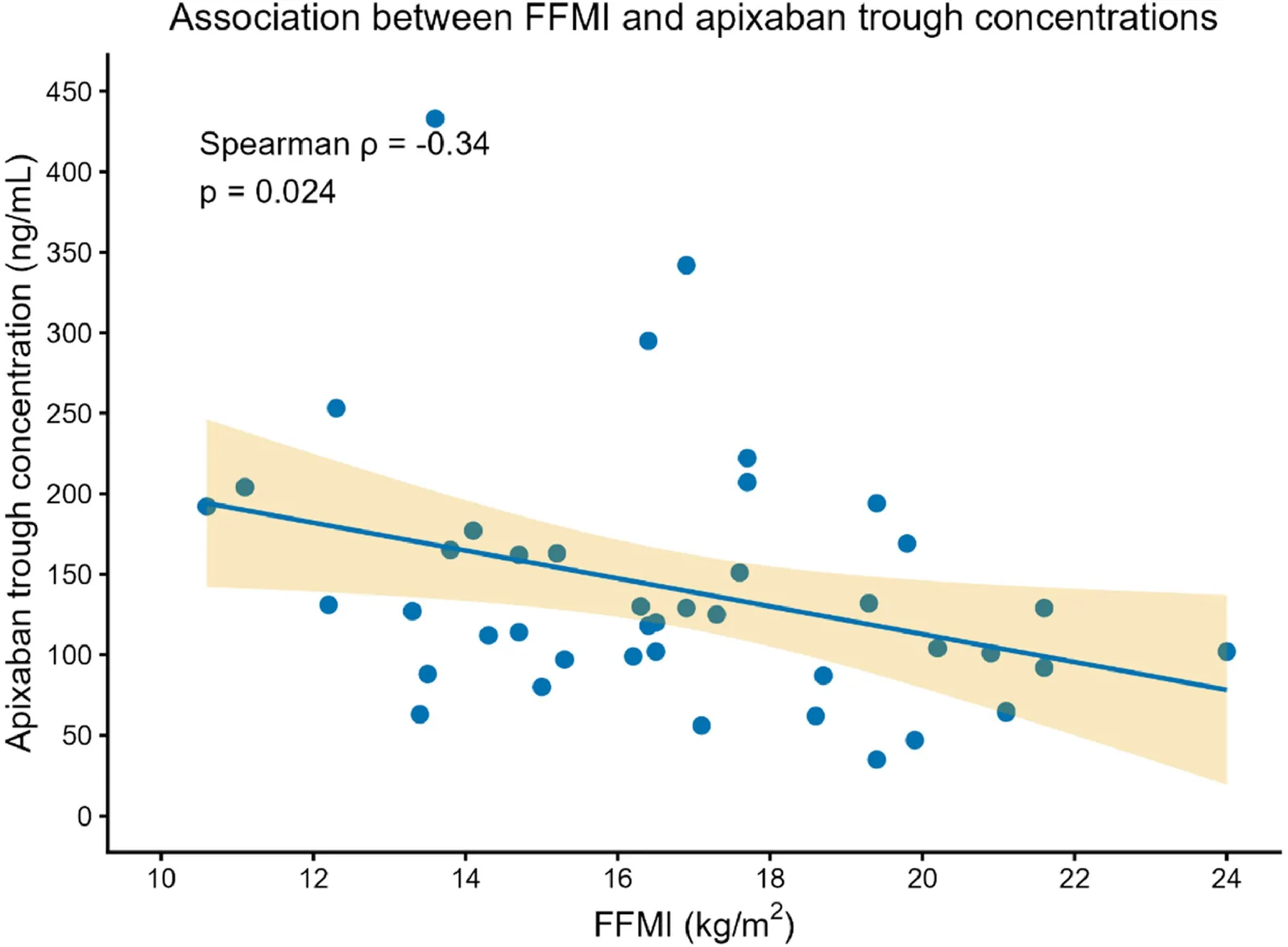
Association between fat-free mass index (FFMI) and apixaban trough concentrations in older hospitalized patients receiving apixaban 5 mg twice daily.

An inverse correlation was observed between FFMI and apixaban trough concentrations in the primary analysis population receiving apixaban 5 mg twice daily (Spearman’s ρ = −0.34, *p* = 0.024) (Fig. 2). Substantial interindividual variability and overlap in apixaban concentrations were observed across the range of FFMI values.

The blue line represents the fitted linear regression line, and the shaded area represents its 95% confidence interval. The Spearman correlation coefficient and corresponding *p*-value are displayed in the figure. FFMI: fat-free mass index.

In univariable linear regression analyses, lower FFMI was significantly associated with higher apixaban concentrations (β = −8.64 ng/mL per kg/m², 95% CI −16.15 to −1.13; *p* = 0.025). Female sex showed a non-significant trend toward higher concentrations (β = 47.61 ng/mL, 95% CI −1.85 to 97.07; *p* = 0.059). Age ≥85 years, body weight, serum creatinine, frailty, and severe undernutrition were not significantly associated with apixaban concentrations (Table 2).

**Table 2:** Univariable and multivariable linear regression analyses of factors associated with apixaban trough concentrations in the primary analysis population (*n* = 43).

	Apixaban trough concentrations (ng/mL)			
	β [95% CI]	*P*-value	Adjusted β [95% CI]	Adjusted *P*-value
Female sex	47.61 [−1.85; 97.07]	0.059	30.04 [−23.62; 83.70]	0.264
Age ≥85 years	31.50 [−17.44; 80.44]	0.201		
FFMI (kg/m²)	−8.64 [−16.15; −1.13]	**0.025**	−6.77 [−15.20; 1.65]	0.112
Serum creatinine (µmol/L)	−0.18 [−1.19; 0.84]	0.728	−0.01 [−0.99; 0.98]	0.990
Weight (kg)	−0.16 [−1.69; 1.37]	0.832		
CFS	−4.95 [−28.95; 19.05]	0.679		
Severe undernutrition	9.82 [−39.69; 59.33]	0.691		

β coefficients from univariable and multivariable linear regression models represent the estimated effect of each independent variable on trough apixaban concentration (ng/mL). Values are presented with 95% confidence intervals (95% CI) and corresponding *p*-values. The multivariable model included FFMI, sex, and serum creatinine, which were selected a priori based on biological plausibility and clinical relevance. β: regression coefficient (unadjusted or adjusted as specified); FFMI: Fat Free Mass Index; CFS: Clinical Frailty Scale. Severe undernutrition was operationally defined as serum albumin <30 g/L.

In the multivariable model including sex, FFMI, and serum creatinine, FFMI was not significantly associated with apixaban concentrations (adjusted β = −6.77 ng/mL per kg/m², 95% CI −15.20 to 1.65; *p* = 0.112); female sex and serum creatinine were also not significant.

In sensitivity analyses including patients receiving both apixaban 5 mg and 2.5 mg twice daily, the correlation between FFMI and apixaban trough concentrations was weaker and no longer statistically significant (*n* = 82; Spearman’s ρ = −0.06, *p* = 0.575). No significant association was observed in the 2.5-mg group considered separately (*n* = 39; ρ = 0.14, *p* = 0.391).

### 3.4 High apixaban exposure

Eleven patients had trough concentrations in the upper observed quartile (≥167 ng/mL), and four patients (9.3%) had concentrations above the upper expected on-therapy trough range (>230 ng/mL) (Table 3). Patients in the upper observed quartile had a lower mean FFMI than the remaining population (15.4 ± 3.2 vs 17.3 ± 3.0 kg/m²), although the difference was not statistically significant (*p* = 0.181). Weight, BMI, and CKD-EPI 2021 estimates were also not significantly different between groups. Female sex was more frequent among patients with higher concentrations, but this difference was not statistically significant (54.5%vs 28.1%; *p* = 0.150).

**Table 3:** Comparison between patients with higher apixaban exposure and the remaining study population (*n* = 43).

	Higher exposure (≥167.0 ng/mL), *n* = 11	Remaining population *n* = 32	*P*-value
Age, years	84.0 ± 5.9	83.4 ± 5.9	0.802
Female sex, n (%)	6 (54.5%)	9 (28.1%)	0.150
Weight, kg	74.4 ± 16.3	76.7 ± 16.5	0.738
BMI, kg/m²	28.1 ± 7.8	27.5 ± 6.1	1.000
FFMI, kg/m²	15.4 ± 3.2	17.3 ± 3.0	0.181
CKD-EPI 2021, mL/min/1.73 m²	76.7 ± 22.4	80.6 ± 14.2	0.878

BMI: body mass index; CKD-EPI: Chronic Kidney Disease Epidemiology Collaboration; eGFR: estimated glomerular filtration rate; FFMI: fat-free mass index.

### 3.5 Renal function estimation and apixaban exposure

None of the evaluated creatinine-based renal estimates was significantly correlated with apixaban trough concentrations (Supplementary Table 1). Cockcroft–Gault estimates were moderately correlated with FFMI (Spearman’s ρ = 0.44, nominal *p* = 0.003), whereas correlations between FFMI and the other renal estimates were weak and not statistically significant.

## 4 Discussion

In this exploratory study of frail hospitalized patients receiving apixaban, an inverse correlation between FFMI and apixaban trough concentrations was observed in the unadjusted analysis restricted to patients receiving apixaban 5 mg twice daily. This signal was attenuated and no longer statistically significant after multivariable adjustment or in the sensitivity analysis including both dose groups. These findings therefore do not establish FFMI as an independent determinant of apixaban exposure and should be considered hypothesis-generating. Body weight, BMI, and conventional creatinine-based estimates of renal function were not significantly associated with apixaban concentrations.

The unadjusted inverse relationship between FFMI and apixaban exposure has a biologically plausible basis. Reduced lean mass may lower creatinine production and lead creatinine-based equations to overestimate renal function [21], while a lower apparent volume of distribution could theoretically increase plasma concentrations. Bendayan et al. similarly reported higher DOAC activity in older adults with lower lean mass [6]. Although our unadjusted findings are consistent with this biological hypothesis, their lack of persistence after adjustment and in sensitivity analysis prevents any conclusion regarding an independent effect of FFMI on apixaban exposure. However, neither renal clearance nor volume of distribution was directly measured, and no pharmacokinetic modelling was performed. Moreover, none of the creatinine-based equations correlated significantly with apixaban trough concentrations, although Cockcroft–Gault was correlated with FFMI. These findings do not establish that renal equations inadequately predict apixaban exposure or that FFMI independently determines it. Previous studies have shown that lean mass is more strongly associated with serum and urinary creatinine than with cystatin C, supporting the concept that creatinine-based renal assessment may be biased in patients with altered body composition [21].

Body weight and BMI were not significantly associated with apixaban concentrations, but the present findings do not demonstrate that FFMI provides additional predictive value. Similar body weight or BMI values may nevertheless reflect different body-composition profiles in frail older adults, particularly in sarcopenia, fluid imbalance, or sarcopenic obesity [22]. Four patients (9.3%) had trough concentrations above 230 ng/mL, the upper boundary of an expected on-therapy range rather than a validated toxicity threshold. Direct comparison with previous reports is limited by differences in populations, dosing regimens, assays, and sampling conditions [19,20].

For stroke prevention in atrial fibrillation, current apixaban dose-adjustment strategies mainly rely on age, body weight, and serum creatinine [7]. At present, these findings do not support the routine use of FFMI for anticoagulant dose adjustment in clinical practice. Nevertheless, they support further prospective studies integrating body composition assessment, pharmacokinetic analyses, and potentially alternative renal biomarkers such as cystatin C in frail older adults.

This study has several strengths. It specifically focused on a hospitalized geriatric population characterized by advanced age, frailty, and heterogeneous body composition profiles, which remains underrepresented in pharmacokinetic studies of direct oral anticoagulants. In addition, apixaban exposure was assessed using measured trough plasma concentrations obtained in routine clinical practice, and body composition was evaluated using bioelectrical impedance analysis with calculation of FFMI.

However, several limitations should be acknowledged. First, the retrospective, single-center design and small sample size limited statistical power, particularly for multivariable and subgroup analyses, and resulted in imprecise estimates with wide confidence intervals. This may have prevented the detection of modest independent associations. Conversely, several exploratory analyses were conducted without adjustment for multiple comparisons, and the nominal *p* = 0.024 observed in the unadjusted primary analysis may represent a chance finding. The results should therefore be interpreted as exploratory and hypothesis-generating.

Second, restricting the primary analysis to patients receiving apixaban 5 mg twice daily reduced heterogeneity directly attributable to dose but also selected a specific clinical subgroup. In routine practice, dose allocation is influenced by age, body weight, renal function, and other clinical characteristics. Accordingly, patients receiving 2.5 mg twice daily differed from those receiving 5 mg twice daily in several respects, including age, sex, FFMI, and renal function. Moreover, the inverse relationship observed in the standard-dose group was not found when both dosing regimens were included. Dose-selection bias, residual confounding, and limited generalizability across dose groups therefore cannot be excluded.

Third, the study population may have been affected by selection bias. A substantial proportion of screened patients could not be included because apixaban concentrations or assessable BIA measurements were unavailable, or because sampling did not meet the predefined trough conditions. Although no statistically significant differences were observed between patients with compliant and non-compliant sampling among the 97 patients with both measurements available, detailed characteristics were not available for the entire screened population. Patients without assessable BIA measurements may have differed systematically from those included, particularly because edema, implanted cardiac devices, or greater clinical instability may be associated with frailty and altered body composition.

Finally, several measurement-related limitations should be considered. FFMI alone does not establish a diagnosis of sarcopenia, which also requires assessment of muscle strength and physical performance. Sampling was operationally classified as compliant when performed at the end of the dosing interval before the next administration or within 30 min after administration. Although based on manually verified source data, this pragmatic definition included some early post-dose samples that may not represent true predose trough concentrations and may have introduced timing-related variability. Only trough concentrations were available, and these do not characterize peak exposure or the overall pharmacokinetic profile. The specific clinical indication for requesting apixaban measurement was not systematically recorded, so indication bias related to the treating team’s decision to monitor drug concentrations cannot be excluded. In addition, cystatin C and other alternative renal biomarkers were unavailable, and no robust bleeding or thromboembolic outcome data were collected, limiting both the mechanistic and clinical interpretation of the findings.

## 5 Conclusion

In older hospitalized patients receiving apixaban 5 mg twice daily, lower FFMI values were associated with higher apixaban trough concentrations in the unadjusted analysis, whereas body weight and BMI were not associated with drug exposure. However, the association with FFMI was not confirmed after multivariable adjustment or in the sensitivity analysis including both dose groups. Conventional creatinine-based estimates of renal function also showed limited correlation with apixaban concentrations in this frail geriatric population. Although exploratory and requiring confirmation in larger prospective studies, these findings raise the hypothesis that body composition may contribute to apixaban exposure variability, but they do not establish FFMI as an independent predictor. Further prospective studies integrating body composition assessment, pharmacokinetic analyses, and alternative renal biomarkers are needed to better characterize anticoagulant exposure in frail geriatric patients.

## Funding

This research did not receive any specific grant from funding agencies in the public, commercial, or non-profit sectors.

## Declaration of the use of generative AI and AI-assisted technologies IN scientific writing and in figures, images and artwork

During the preparation of this manuscript, the authors used ChatGPT (OpenAI) to assist with text refinement and improvement of readability. No generative AI was used to generate, alter, or fabricate primary research data. The authors take full responsibility for the content of the publication.

## CRediT authorship contribution statement

**Tancrède de Jerphanion:** Writing – original draft, Investigation, Formal analysis, Data curation. **Olivier Brière:** Writing – review & editing, Supervision, Methodology, Conceptualization. **Jennifer Gautier:** Writing – review & editing, Formal analysis. **Alexis Bourgeais:** Writing – review & editing, Investigation, Data curation. **Myriam Mellou:** Writing – review & editing, Investigation, Data curation. **Léa Bourreau:** Writing – review & editing, Investigation, Data curation. **Cédric Annweiler:** Writing – review & editing, Supervision, Methodology, Conceptualization. **Mathieu Corvaisier:** Writing – review & editing, Supervision, Methodology, Conceptualization.

## Declaration of competing interest

The authors have declared no conflict of interest

## Data Availability

The datasets generated and analyzed during the current study are not publicly available due to patient confidentiality but are available from the corresponding author upon reasonable request.

## Appendix Supplementary materials

Image, application 1

Image, application 2

Image, application 3
