# Supplementary material for: Fat-free mass index and apixaban exposure in frail hospitalized older adults: An exploratory study
Source: J Frailty Aging. 2026 Sep 3;15(5):100201. doi: 10.1016/j.tjfa.2026.100201 (PMC13571488; doi:10.1016/j.tjfa.2026.100201)
Supplement: Supplementary file 1 [file mmc1.docx]

**Supplementary Table 1**. Correlations between renal function estimations, FFMI, and apixaban trough concentrations in the primary analysis population (n = 43)

|  | **Correlation with apixaban trough concentration (Spearman’s ρ)** | **P-value** | **Correlation with FFMI (Spearman’s ρ)** | **P-value** |
| --- | --- | --- | --- | --- |
| CKD-EPI 2021 | -0.128 | 0.412 | 0.034 | 0.828 |
| MDRD | -0.076 | 0.628 | -0.057 | 0.715 |
| Cockcroft–Gault | -0.177 | 0.256 | 0.444 | **0.003** |
| BIS-1 | -0.137 | 0.382 | 0.025 | 0.874 |

*BIS-1: Berlin Initiative Study 1 equation; CKD-EPI: Chronic Kidney Disease Epidemiology Collaboration; FFMI: fat-free mass index; MDRD: Modification of Diet in Renal Disease.*
