# Supplementary material for: Fat-free mass index and apixaban exposure in frail hospitalized older adults: An exploratory study
Source: J Frailty Aging. 2026 Sep 3;15(5):100201. doi: 10.1016/j.tjfa.2026.100201 (PMC13571488; doi:10.1016/j.tjfa.2026.100201)
Supplement: Supplementary file 2 [file mmc2.docx]

**Supplementary Table 2**. Comparison of baseline characteristics according to trough sampling compliance among patients with both apixaban concentration and bioelectrical impedance measurements available (n = 97)

|  |  |  |  |
| --- | --- | --- | --- |
|  | **Compliant sampling**  **(n = 82)** | **Non-compliant sampling**  **(n = 15)** | **P-value** |
| Age, years | 86.4 ± 6.1 | 85.5 ± 5.5 | 0.463 |
| Female sex, n (%) | 44 (53.7%) | 6 (40.0%) | 0.405 |
| Apixaban 5 mg BID, n (%) | 43 (52.4%) | 8 (53.3%) | 1.000 |
| Clinical Frailty Scale | 5.4 ± 0.9 | 5.5 ± 1.0 | 0.646 |
| Charlson Comorbidity Index | 8.8 ± 2.9 | 10.1 ± 2.6 | 0.086 |
| ADL score | 4.0 ± 1.6 | 3.3 ± 2.1 | 0.259 |
| IADL score | 1.4 ± 1.2 | 1.2 ± 1.5 | 0.502 |
| Weight, kg | 68.5 ± 17.4 | 69.7 ± 18.3 | 0.850 |
| BMI, kg/m² | 25.9 ± 6.2 | 25.7 ± 5.4 | 0.818 |
| FFMI, kg/m² | 15.3 ± 3.5 | 15.9 ± 4.3 | 0.791 |
| Serum creatinine, µmol/L | 82.4 ± 30.1 | 96.9 ± 38.1 | 0.110 |
| CKD-EPI 2021, mL/min/1.73 m² | 71.1 ± 20.1 | 64.3 ± 20.1 | 0.193 |
| Serum albumin, g/L | 30.2 ± 5.0 | 27.9 ± 4.8 | 0.115 |

*Values are presented as mean ± standard deviation or n (%). P-values were calculated using the Mann–Whitney U test for continuous variables and Fisher’s exact test for categorical variables. Sampling compliance was classified using the same operational rule as in the primary and sensitivity analyses. ADL: Activities of Daily Living; BIA: bioelectrical impedance analysis; BID: twice daily; BMI: body mass index; CKD-EPI: Chronic Kidney Disease Epidemiology Collaboration; eGFR: estimated glomerular filtration rate; FFMI: fat-free mass index; IADL: Instrumental Activities of Daily Living.*
