# Supplementary material for: Fat-free mass index and apixaban exposure in frail hospitalized older adults: An exploratory study
Source: J Frailty Aging. 2026 Sep 3;15(5):100201. doi: 10.1016/j.tjfa.2026.100201 (PMC13571488; doi:10.1016/j.tjfa.2026.100201)
Supplement: Supplementary file 3 [file mmc3.docx]

**Supplementary Table 3.** Comparison of characteristics according to apixaban dosing regimen among patients with compliant trough sampling (n = 82)

|  |  |  |  |
| --- | --- | --- | --- |
|  | **Apixaban 5 mg twice daily (n = 43)** | **Apixaban 2.5 mg twice daily (n = 39)** | **P-value** |
| Age, years | 83.6 ± 5.8 | 89.4 ± 4.8 | **<0.001** |
| Female sex, n (%) | 15 (34.9%) | 29 (74.4%) | **<0.001** |
| Clinical Frailty Scale | 5.2 ± 1.0 | 5.7 ± 0.7 | **0.039** |
| Charlson Comorbidity Index | 7.8 ± 2.4 | 9.9 ± 3.1 | **0.002** |
| ADL score | 4.3 ± 1.6 | 3.7 ± 1.4 | **0.039** |
| IADL score | 1.5 ± 1.4 | 1.2 ± 1.1 | 0.486 |
| Weight, kg | 76.1 ± 16.3 | 60.2 ± 14.7 | **<0.001** |
| BMI, kg/m² | 27.7 ± 6.5 | 23.9 ± 5.3 | **0.010** |
| FFMI, kg/m² | 16.8 ± 3.1 | 13.7 ± 3.2 | **<0.001** |
| Serum creatinine, µmol/L | 75.2 ± 24.5 | 90.4 ± 33.8 | **0.039** |
| CKD-EPI 2021, mL/min/1.73 m² | 79.6 ± 16.4 | 61.8 ± 19.8 | **<0.001** |
| Serum albumin, g/L | 30.2 ± 5.0 | 30.2 ± 5.0 | 0.638 |
| Apixaban trough concentration, ng/mL | 140.5 ± 79.0 | 125.2 ± 81.3 | 0.281 |

*Values are presented as mean ± standard deviation or n (%). P-values were calculated using the Mann–Whitney U test for continuous variables and Fisher’s exact test for categorical variables. ADL: Activities of Daily Living; BIA: bioelectrical impedance analysis; BMI: body mass index; CKD-EPI: Chronic Kidney Disease Epidemiology Collaboration; eGFR: estimated glomerular filtration rate; FFMI: fat-free mass index; IADL: Instrumental Activities of Daily Living.*
